# Aortic Root Abscess with Aorta to Right Atrium Fistula due to Vancomycin-Intermediate Staphylococcus aureus (VISA)

**DOI:** 10.1155/2019/3627063

**Published:** 2019-05-29

**Authors:** Stephen J. Hankinson, Elliot A. Sultanik, Gautam V. Ramani

**Affiliations:** Department of Medicine, Division of Cardiovascular Medicine, University of Maryland School of Medicine, Baltimore, Maryland 21201, USA

## Abstract

We present a case of root abscess with aorta to right atrium fistula due to vancomycin-intermediate Staphylococcus aureus (VISA) after limb amputation and cardiac surgery. Patient underwent redo aortic valve replacement, patch repair of aorta to right atrial fistula, and tricuspid valve repair with a ring. Fistula formation is a rare complication of prosthetic valve endocarditis (PVE). This is the first case to discuss aortocavitary fistula (ACF) formation due to VISA. Transesophageal echocardiogram (TEE) is the preferred imaging modality to diagnose ACF.

## 1. Introduction

The emergence of multidrug-resistant (MDR) organisms makes the medical management of endocarditis increasingly difficult. TEE is the preferred imaging modality to diagnose ACF. Surgical repair continues to be the definitive treatment option for ACF.

## 2. Case Presentation

Our patient is a 74-year-old man with a history of rheumatoid arthritis (on prednisone), left below the knee amputation (BKA), coronary artery bypass graft (CABG) with a saphenous vein graft to the right coronary artery (SVG to RCA), aortic valve replacement (AVR) with a bioprosthetic valve, and mitral valve replacement (MVR) with a bioprosthetic valve who presented with fever, chills, and generalized weakness after a prolonged course of vancomycin for methicillin-resistant Staphylococcus aureus (MRSA) bacteremia.

**Figure 1:**
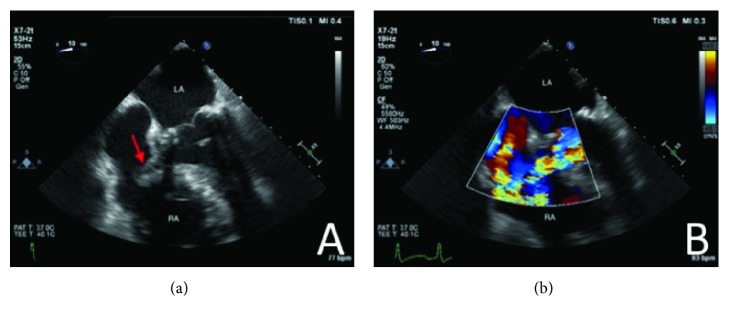
TEE demonstrates mobile echodensity (red arrow) measuring 2.4 cm × 1.9 cm in the RA associated with the wall of the atrium at the site of the suspected defect (a). TEE demonstrates atrioventricular defect and a systolic left to right shunt suggestive of aortic root abscess and erosion into the RA (b).

The patient was previously admitted for left foot MRSA osteomyelitis. During that hospitalization, the patient had a myocardial infarction. Source control obtained with BKA of the left leg and five days later subsequently underwent CABG (SVG to RCA), AVR for severe aortic stenosis (AS), and MVR for severe mitral regurgitation (MR). The patient was discharged with a six-week course of vancomycin; however, vancomycin course was extended due to BKA wound that required skin graft surgery. Eighty-eight days after cardiac surgery, the patient was readmitted for MRSA bacteremia attributed to cellulitis of the BKA stump vs. endocarditis. Transthoracic echocardiogram (TTE) at that time was negative for PVE. Patient was treated with a six-week course of vancomycin, and BKA stump cellulitis subsequently resolved. Five days after completing antibiotics, the patient presented with MRSA bacteremia, which progressed to VISA bacteremia with a vancomycin minimum inhibitory concentration (MIC) of 4 *μ*g/mL (Table 1). The patient was started on daptomycin, ceftaroline, and rifampin for VISA bacteremia. TEE showed aortic root thickening suggestive of abscess with associated structural defect, a mobile 2.4 cm × 1.9 cm echodensity projecting into the right atrium (RA) (Figure 1(a)), and left to right shunting (Figure 1(b)).

The patient subsequently underwent cardiac surgery for redo AVR with a bovine pericardial tissue valve, patch repair of aorta to right atrial fistula with a bovine pericardial patching material from both the left ventricular side and right atrial side, and tricuspid valve repair with a ring. Postsurgery TTE demonstrated a normal left ventricle size and systolic function, a normal right ventricle size and function, a bioprosthetic valve in aortic position (mean gradient 20 mmHg and peak gradient 45 mmHg) and no aortic regurgitation, and an annuloplasty ring indicative of tricuspid valve repair.

## 3. Discussion

This case discusses ACF formation as a complication of cardiac surgery shortly after limb amputation. PVE is a devastating complication of valve replacement with the prevalence being 4.1% at 4 years after primary valve replacement with the greatest risk occurring 1 to 2 months after surgery [1].

ACF is a rare condition caused by congenital abnormalities, aortic dissection, valve replacement, and infective endocarditis (IE) [2]. The pathogenesis of ACF related to infection is caused by extension and infiltration of abscesses related to endocarditis. Annular infection affects contiguous tissue leading to pyogenesis and tissue necrosis, which causes formation of an abscess cavity. As a result, the weakened necrotic myocardium may expand and rupture which may create intracardiac fistulous communications or even pericardial shunts [3]. Echocardiography can be used to identify the location of the lesion while angiography is often used to demonstrate the course of the lesion and define the surgical approach. Anguera et al. demonstrated that the detection of ACF for TTE and TEE is 53% and 97%, respectively [4]. TEE is the superior imaging technique because the flow across the fistula is highly turbulent and Doppler mapping can easily detect pressure differences between the aorta and cardiac chambers even when the fistulous orifice is small; therefore, TEE allows for the detection of almost all fistulas and allows the optimal characterization of each fistula tract. Additionally, TEE is superior to TTE in assessing valve function and morphology as well as delineating intracardiac pathology such as complications of endocarditis, namely, root abscess and fistulas. Furthermore, TEE has a better signal to the noise ratio and proximity of the transducer to the heart leading to higher quality images with lesser attenuation [5].

Our case is unique because it is the first case of ACF due to VISA. Our patient had extensive comorbidities prior to BKA including cardiac issues, sepsis, and steroid use, all of which have been shown to be predictors of increased mortality and postoperative complications within 30 days after BKA [6]. Given these comorbidities, perhaps a longer time course should have elapsed between the BKA in order to further optimize the patient prior to cardiac surgery. Overall, these comorbidities lead to a prolonged course of vancomycin, which resulted in the development of a MDR organism.

VISA and vancomycin-resistant S. aureus (VRSA) are relatively rare infections that occur in the setting of heavy prior use of glycopeptide antibiotics [7]. The Clinical Laboratory Standards Institute (CLSI) defines VISA as a vancomycin MIC of 4-8 *μ*g/mL and VRSA as a vancomycin MIC of ≥16 μg/mL [8]. Vancomycin MIC ≥ 4 *μ*g/mL is associated with vancomycin treatment failures in MRSA IE, which is a novel concept for this cohort [9]. MRSA resistance to vancomycin occurs through a variety of mechanisms including cell wall thickening, decreased autolysis, reduced production of cell surface protein A, increased capsule expressions, increased D-alanylation of teichoic acids, and reduced *agr* activity [8]. Given the prolonged course of vancomycin prior to the discovery of the ACF, it is difficult to identify whether the valve annulus was seeded during implantation of the valve or BKA stump cellulitis lead to hematogenous seeding of the valve.

## 4. Conclusion

Presented above was a case of a patient with an ACF due to VISA. This case demonstrated that protracted use of vancomycin resulted in VISA IE. Clinicians should exercise caution in patients with high bacterial load infections after prolonged antibiotic use. Infectious disease specialist consultation, change in antibiotic regimen, and aggressive surgical management are appropriate for the management of VISA IE.

## Figures and Tables

**Table 1:** Culture data during protracted use of vancomycin.

Type of culture	Date	Organism	Vancomycin MIC (*μ*g/mL)
Left foot wound	11/10/2017	MRSA	≤0.5
Blood	2/27/2018	MRSA	1
Blood	4/16/2018	MRSA	2
Blood	4/26/2018	MRSA	4

## Supplementary Materials

Supplementary Materials “Color doppler video” (supplementary material 1) 2.4 cm × 1.9 cm mobile echodensity in the RA with a systolic left to right shunt.
